# Automated expiratory ventilation assistance through a small endotracheal tube can improve venous return and cardiac output

**DOI:** 10.1186/s40635-018-0217-y

**Published:** 2019-01-09

**Authors:** David A. Berlin, Seth Manoach, Clara Oromendia, Paul M. Heerdt

**Affiliations:** 1 Division of Pulmonary and Critical Care Medicine, Weill Cornell Medicine, 1300 York Avenue, New York, NY 10065 USA; 2 Division of Biostatistics and Epidemiology, Weill Cornell Medicine, 1300 York Avenue, New York, NY 10065 USA; 3 Division of Applied Hemodynamics, Yale University School of Medicine, 333 Cedar St, P.O. BOX 208051, New Haven, CT 06520-8051 USA

**Keywords:** Cardiac output, Venous return, Negative pressure ventilation, Hemorrhage, Expiratory ventilation assistance

## Abstract

**Background:**

Positive pressure ventilation can decrease venous return and cardiac output. It is not known if expiratory ventilation assistance (EVA) through a small endotracheal tube can improve venous return and cardiac output.

**Results:**

In a porcine model, switching from conventional positive pressure ventilation to (EVA) with − 8 cmH_2_0 expiratory pressure increased the venous return and cardiac output. The stroke volume increased by 27% when the subjects were switched from conventional ventilation to EVA [53.8 ± 7.7 (SD) vs. 68.1 ± 7.7 ml, *p* = 0.003]. After hemorrhage, subjects treated with EVA had higher median cardiac output, higher mean systemic arterial pressure, and lower central venous pressure at 40 and 60 min when compared with subjects treated with conventional ventilation with PEEP 0 cmH_2_0. The median cardiac output was 41% higher in the EVA group than the control group at 60 min [2.70 vs. 1.59 L/min, *p =* 0.029].

**Conclusion:**

EVA through a small endotracheal tube increased venous return, cardiac output, and mean arterial pressure compared with conventional positive pressure ventilation. The effects were most significant during hypovolemia from hemorrhage. EVA provided less effective ventilation than conventional positive pressure ventilation.

## Background

Positive pressure ventilation can reduce cardiac output by impeding venous return to the heart. The most significant reduction occurs in hypovolemic patients, especially if the ventilator maintains positive pressure during both inspiration and exhalation [1–4]. Conversely, negative pressure ventilation can improve cardiac output by reducing intrathoracic pressure and increasing the pressure gradient for venous return to the heart [5–7]. Alternating positive pressure inspiration with negative pressure during exhalation increases cardiac output when compared with strategies using positive end-expiratory pressure (PEEP) [6, 8–10]. In laboratory models of hemorrhage shock, negative pressure ventilation can restore cardiac output [9, 11]. These findings suggest a possible benefit of negative expiratory pressure as part of the initial management of patients with hemorrhagic shock who require mechanical ventilation. However, there are some potential problems with this approach. First, negative intrathoracic pressure can collapse the great veins and limit venous return [7]. Additionally, negative intrathoracic pressure may increase the risk of pulmonary edema or atelectasis [12–14]. Finally, it is unclear how the variety of different negative pressure techniques differs from one another. Therefore, more information is needed on the effects of negative pressure exhalation strategies in hemorrhagic shock.

**Fig. 1:**
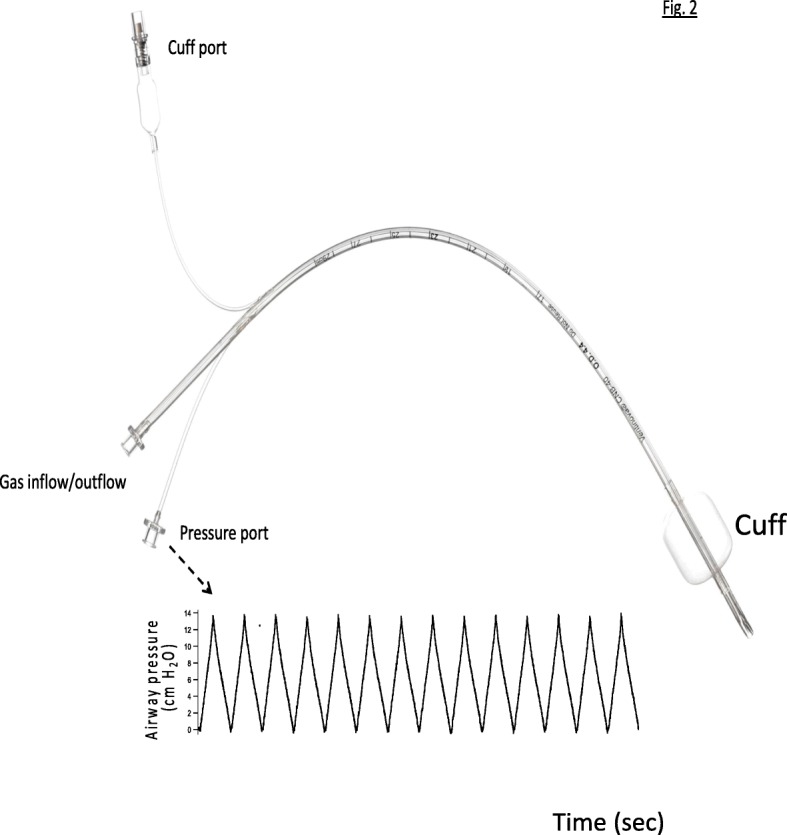
The Tritube used for expiratory ventilation assistance has three ports on the proximal end. An example of the pressure-time tracing from the airway is shown at the bottom. The end-expiratory pressure has been set to 0 cmH_2_O

Recently, a mechanical ventilation device that creates negative intratracheal pressure during exhalation became available (Ventrain, Ventinova Medical BV, Eindhoven, NL). Ventrain uses positive pressure during inspiration, and a technique called expiratory ventilation assistance (EVA) during exhalation. By increasing the efficiency of ventilation, EVA enables the use of an extremely narrow endotracheal catheter. The current indication for this hand-held device is for rescue transtracheal ventilation in a “can’t intubate and can’t ventilate” situation. During inspiration, gas flows through the Ventrain and then into the endotracheal catheter. During exhalation, gas flow through the Ventrain is diverted out a narrowed side port. The diversion creates a Venturi effect—the air pressure in the Ventrain decreases as gas accelerates through the narrowed side port. The lower pressure in the Ventrain facilitates expiratory flow out the endotracheal catheter. With the original hand-held Ventrain, the operator manually controls the duration of inspiration and exhalation [15, 16]. A newly automated modification of Ventrain controls the timing and pressure targets during inspiration and exhalation. The automated device interfaces with a new catheter (Tritube Fig. 1) that has a distal cuff for inflation as well as lumens for side-stream capnography and pressure monitoring. It is unknown if a reduction of the expiratory pressure in the trachea with an EVA system can reduce intrathoracic pressure sufficiently to restore hemodynamics.

The purpose of this study was to determine whether EVA through a small endotracheal tube could improve cardiac output, venous return, and mean arterial pressure compared with conventional mechanical ventilation. To answer this question, we used a porcine model of hemorrhage and resuscitation. The study also compared the effects of the two ventilation strategies on gas exchange.

## Methods

The study was performed along with another study comparing cardiac output monitors in hemorrhagic shock, which has been previously reported [17].

### Experimental preparation

Eight Yorkshire swine (46–55 kg) were sedated with tiletamine/zolazepam/xylazine and initially anesthetized with isoflurane by nose cone. The trachea was intubated with an 8.0 cuffed endotracheal tube and anesthesia converted to propofol/fentanyl/midazolam infusion and rocuronium. Bipolar electroencephalogram (Cerebral State Monitor; Danmeter, Odense, Denmark) was used to monitor the depth of anesthesia. Baseline conventional positive pressure ventilation (volume control) was initiated with a tidal volume of 9 cm^3^/kg, a rate of 12–13, positive end-expiratory pressure (PEEP) of 4 cmH_2_O, and a fraction of inspired oxygen (FiO_2_) of 0.4. A catheter with a ~ 4 cmH_2_O inflatable cuff was positioned in the mid-esophagus to estimate trends in intrathoracic pressure. The zero reference was the pressure within the cuff during a period of apnea with the endotracheal tube open to atmospheric pressure. The femoral arteries and veins were percutaneously cannulated with 9 Fr. vascular sheaths for hemodynamic monitoring. One venous sheath was used for placement of a pulmonary artery catheter to measure PA and central venous pressures as well as serial cardiac output by bolus thermodilution (the average of three injections of 10 cm^3^ 0.9% NaCl at room temperature). The other venous sheath was used for drug and fluid administration. Arterial blood was withdrawn from one arterial sheath and the systemic arterial pressure measured from the side-arm of the other. Via one arterial sheath, a 5 Fr. conductance/micromanometer catheter was advanced into the left ventricle. The parallel conductance of the system was determined by injection of hypertonic saline, and bolus thermodilution served as the reference for calibration of stroke volume. To provide continuous assessment of cardiac output and intrathoracic fluid content, surface sensors for bioreactance monitoring (NICOM, Cheetah Medical Inc. Portland, USA) were placed across the chest. A heating blanket maintained body temperature, and lactated Ringers solution infused at 2 cm^3^/kg/h. All data were time stamped and continuously recorded in digital format by LabChart (ADInstruments, New South Wales, Australia) and the NICOM monitor.

### EVA ventilation

After instrumentation and stabilization, a trial of EVA using a 4.4 mm outer diameter, cuffed intratracheal catheter (Tritube) was performed to assure proper function of the system and determine hemodynamic effects in normovolemia in six of the subjects. The Tritube has three lumens: a 2.3 mm internal diameter lumen for ventilation, a distal port for monitoring intratracheal pressure, and a lumen for inflation/deflation of the cuff (Fig. 1). The Tritube was inserted through the endotracheal tube and the distal tip positioned above the tracheal origin of the right upper (cranial) lobe bronchus with bronchoscopic guidance. The Tritube was marked to assure equal depth of placement each time, and the inflated cuff created a closed system. Ventilation was initiated with an automated Ventrain device. During inspiration, the device delivers a constant rate of gas flow until the pressure measured at the end of the Tritube reaches a limit set by the user. The user also sets the ratio of inspiratory to expiratory times and the desired level of end-expiratory pressure. The EVA ventilator assists expiratory flow by producing a linear decline in intratracheal pressure until reaching the target end-expiratory pressure (Fig. 1). To confirm that the desired levels of negative end-expiratory pressure could be achieved, the target was progressively reduced from + 4 to − 8 cmH_2_O with minute ventilation kept constant. Each step lasted for ~ 8 min with FiO_2_ of 1.0, and arterial blood gases measured. Afterward, the Tritube was removed, and conventional positive pressure ventilation with 4 cmH_2_0 PEEP resumed.

### Hemorrhage and ventilation

During conventional positive pressure ventilation, 40 ml/kg of blood was withdrawn from all eight subjects into heparinized bags over 30 min. Half the subjects continued on conventional positive pressure ventilation (9 cm^3^/kg, rate 12–13) with 0 cmH_2_0 PEEP, FiO_2_ 1.0. The other four subjects were placed on EVA after reinsertion of the Tritube. In the four subjects on EVA, the end-expiratory pressure was progressively reduced over 10 min to − 8 cmH_2_O. One of the four EVA subjects had the end-expiratory pressure reduced to − 10 cmH_2_0. During EVA, FiO_2_ was kept at 1.0 and minute ventilation was adjusted by varying the peak inspiratory pressure, inspiratory flow rate, and the inspiratory to expiratory ratio. EVA was adjusted to continue the same minute ventilation used during conventional ventilation. Arterial blood gas analysis was performed at baseline (pre-hemorrhage), the end of hemorrhage (0 time, during conventional ventilation for both groups), and then at 20-min intervals during shock. After 60 min, all subjects underwent a 20-min resuscitation with a combination of shed blood and 5 cm^3^/kg of warmed lactated Ringers solution. Following resuscitation, the Tritube was removed in those receiving ventilation with EVA. For all animals, a recruitment maneuver of 40 cmH_2_0 for 10 s was performed, and conventional positive pressure ventilation with 4 cmH_2_0 PEEP and FiO_2_ 0.40 continued for another 90 min. Upon conclusion of the protocol, animals were euthanized with intravenous potassium chloride while deeply anesthetized.

### Hemodynamic data recording and analysis

For each subject, the average values for continuous beat-to-beat hemodynamic and breath-to-breath ventilation measurements over three respiratory cycles at each time point were determined, and these data then used along with intermittent bolus thermodilution cardiac output measurements for analysis of pooled data. Measurements across time and treatment within each group were assessed for normality of distribution (Shapiro-Wilk test) and then compared by analysis of variance for repeated measures and the Bonferroni post-hoc test where appropriate. Measurements made during shock and resuscitation were compared independently of those obtained pre-hemorrhage and at 30, 60, and 90 min after resuscitation. This was done to prevent bias of repeated measures analysis by lumping data from the markedly different normovolemic and hypovolemic conditions. Intergroup data (conventional ventilation vs. EVA) at each time point were compared by *t* test. For all analyses, *p* < 0.05 was considered significant. Data are presented as mean + SD.

## Results

**Fig. 2:**
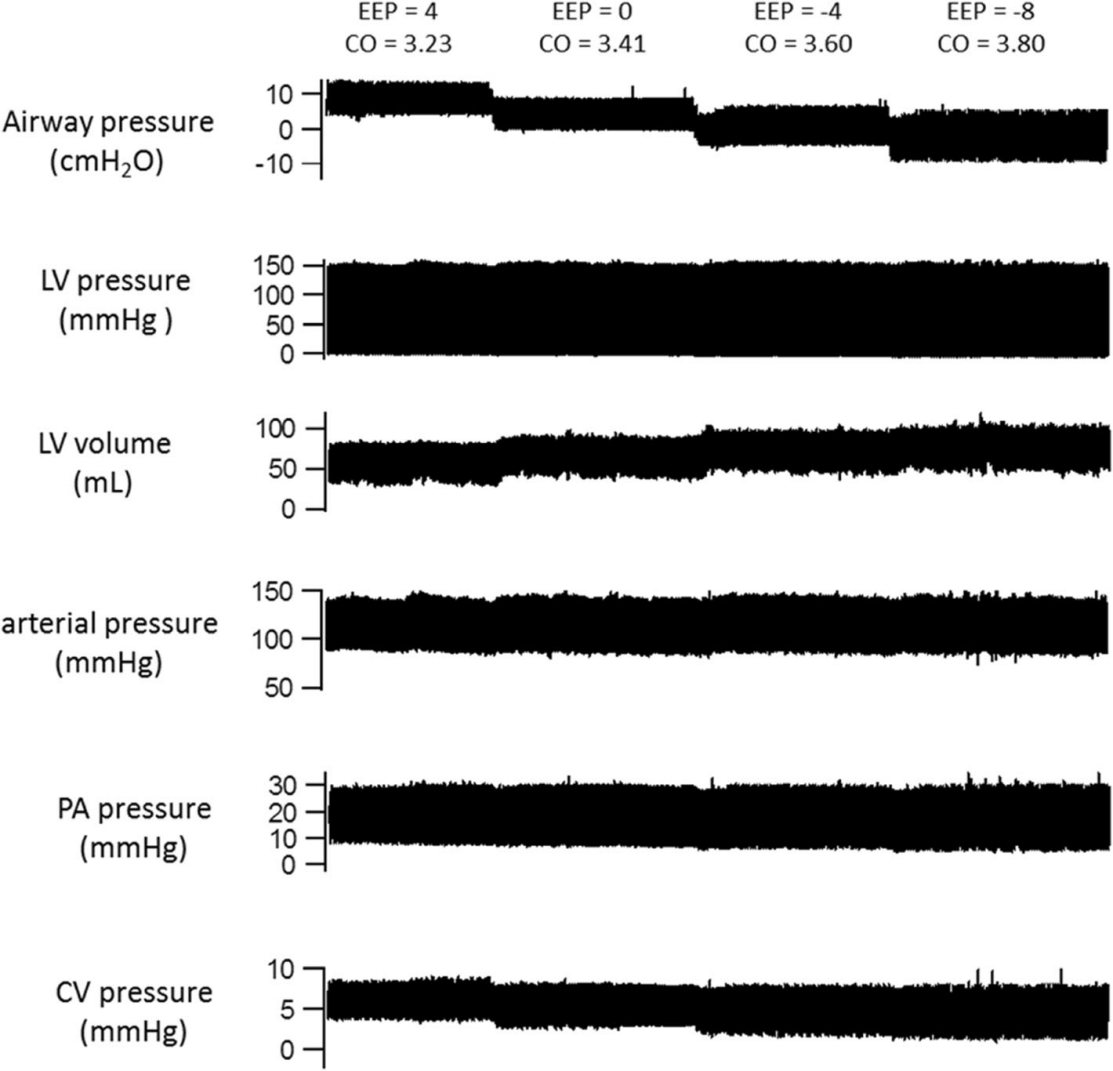
Hemodynamic variables during expiratory ventilation assistance in a normovolemic subject. The end-expiratory pressure (EEP) is decreased over time

Table 1(a) shows the hemodynamic effects before hemorrhage of switching from conventional ventilation with PEEP + 4 cmH_2_O to EVA and then progressively reducing the expiratory pressure to − 8 cmH_2_O. The stroke volume increased by 27% when the subjects switched from conventional ventilation to EVA [53.8 ± 7.7 (SD) vs. 68.1 ± 7.7 ml, *p* = 0.003]. Similarly, the cardiac output increased by 21% [3.53 ± 0.46 (SD) vs. 4.26 ± 0.27 ml, *p* = 0.023]. Additionally, when switched from conventional ventilation to EVA, the central venous pressure fell significantly [6.0 ± 2.0 (SD) vs. 4.2 ± 2.5 mmHg, *p* = 0.013]. There was also a trend toward an increase in left ventricular end-diastolic volume, although this did not reach statistical significance. The progressive hemodynamic changes associated with the stepwise reduction in expiratory pressure during EVA in a single subject are displayed graphically in Fig. 2. As expiratory pressure was reduced from + 4 to − 8 cmH_2_0, the central venous pressure fell while the left ventricular volume and cardiac output rose.

**Table 1:** Effects of expiratory ventilation assistance on physiologic parameters in normovolemia. The end-expiratory pressures for each ventilator condition are shown in the top row. Variables are compared with the values measured at baseline on conventional positive pressure ventilation with 4 cmH_2_0 positive end-expiratory pressure. 1a shows hemodynamic variables and 1b shows gas exchange variables

Variable	Baseline conventional ventilation + 4 cmH_2_0	Expiratory ventilation assistance				Conventional ventilation + 4cmH_2_0
		+ 4 cmH_2_0	0 cmH_2_0	− 4 cmH_2_0	− 8 cmH_2_0	
1a						
Stroke volume (ml)	53.8 (7.7)	58.0 (13.2)	64.6* (10.7)	67.0* (11.7)	68.1* (10.5)	62.3* (12.8)
Cardiac output (L/min)	3.53 (0.46)	3.58 (0.61)	3.86 (0.37)	4.01 (0.30)	4.26* (0.27)	3.54 (0.16)
Left venytricular end-diastolic volume (ml)	87.6 (17.6)	90.0 (17.2)	102.3 (10.8)	102.2 (11.6)	103.8 (10.9)	92.3 (12.9)
Central venous pressure (mmHg)	6.0 (2.0)	5.7 (1.6)	5.3* (1.9)	5.0* (2.0)	4.2* (2.5)	4.4 (2.7)
Mean systemic arterial pressure (mmHg)	110 (10.6)	105 (9.0)	104 (9.1)	105 (10.5)	106 (9.2)	106 (15.5)
Mean pulmonary artery pressure (mmHg)	17.4 (2.5)	17.7 (3.0)	16.8 (2.5)	16.4 (2.5)	16.5 (2.3)	15.5 (2.4)
dP/dt (mmHg/s)	1996 (183)	1979 (173)	1969 (169)	1997 (159)	2039 (160)	1953 (217)
Left ventricular ejection fraction (%)	62.4 (9.0)	64.7 (8.9)	63.2 (8.0)	65.8 (10.0)	66.2 (11.8)	69.7 (10.8)
Heart rate (min^−1^)	65.8 (5.6)	62.7 (8.2)	60.7 (8.6)	61.0 (9.4)	63.9 (11.3)	58.8 (12.8)
1b						
Minute ventilation (L/min)	5.3 (0.3)	5.3 (0.4)	5.3 (0.2)	5.5 (0.5)	5.5 (0.4)	5.2 (0.2)
Peak inspiratory pressure (cmH_2_0)	19.5 (1.8)	13.5* (2.6)	10.5* (2.6)	8.6* (3)	7.0* (3.7)	19 (2.9)
pH	7.48 (0.02)	7.41 (0.04)	7.42 (0.05)	7.42 (0.05)	7.43 (0.07)	7.50 (0.06)
PCO_2_ (mmHg)	44.0 (5.1)	53.4* (8.3)	52.2* (11.0)	51.2* (9.4)	49.8 (12.2)	43.4 (7.5)
PO_2_/FiO_2_	487 (61)	456 (53)	466 (37)	459 (69)	431 (41)	466 (203)

Values are expressed as mean (SD)

*Refers to statistically significant difference from baseline

Table 1(b) shows the gas exchange response to EVA before hemorrhage. EVA resulted in a lower peak inspiratory pressure compared with the baseline condition of conventional positive pressure ventilation with PEEP 4 cmH_2_O. The PCO_2_ was higher during EVA than during conventional ventilation. The right to left shunt fraction, as estimated by the ratio of PaO_2_ to FiO_2_, did not change significantly when switching to EVA [18].

**Fig. 3:**
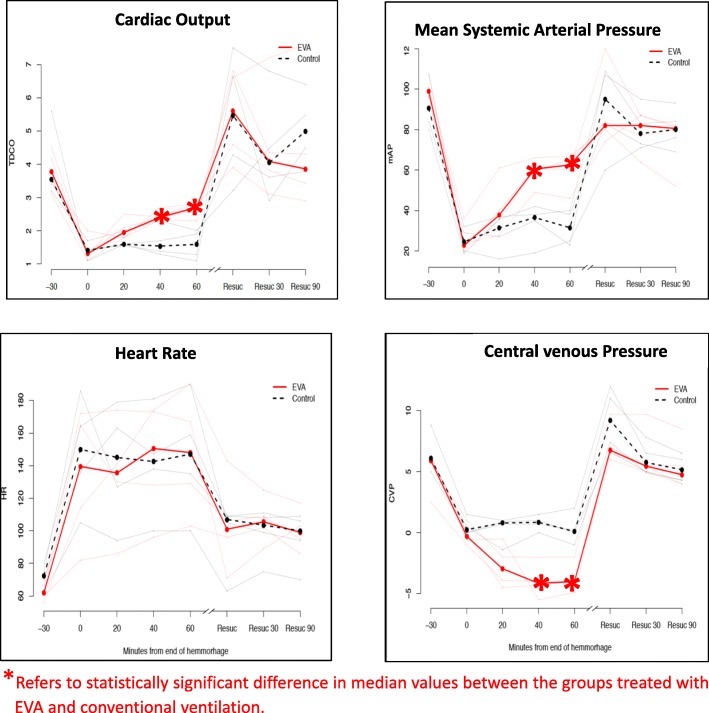
Comparison of expiratory ventilation assistance and conventional ventilation in hypovolemia. All subjects began hemorrhage on conventional ventilation with PEEP 4 cmH_2_0. After completing hemorrhage (time 0 min), the EVA group underwent ventilation with progressively negative end-expiratory pressure. The conventional ventilation group had PEEP 0 cmH_2_0. The solid red and dashed black lines are plots of the median values. The raw data from each of the eight subjects appear in the background. Resuscitation began after measurements at time 60 min

**Fig. 4:**
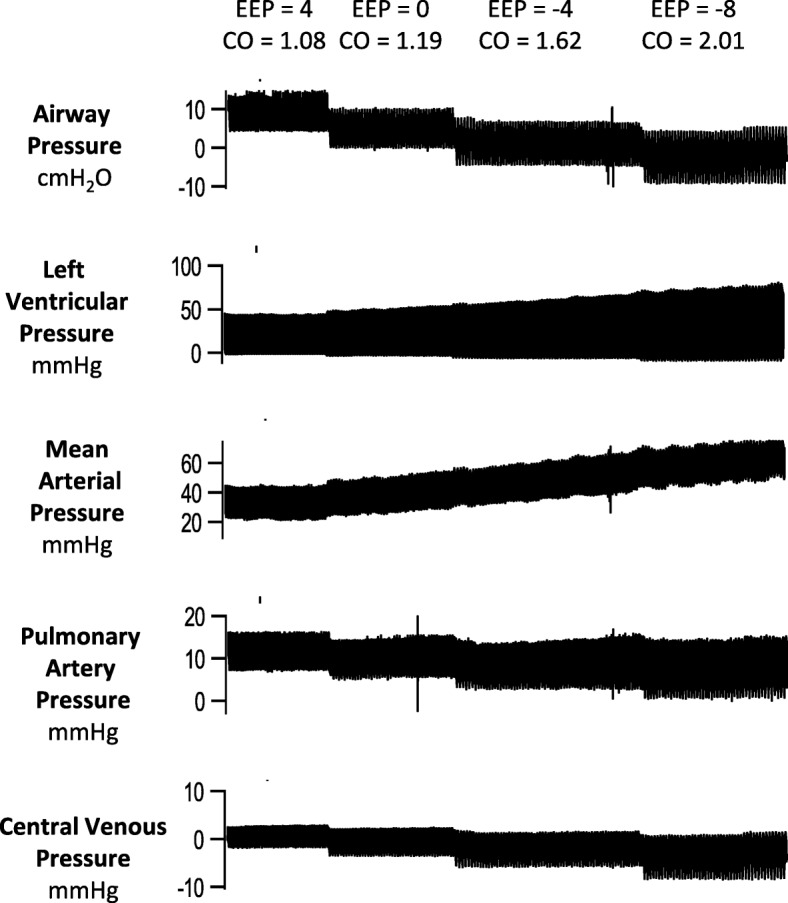
Example of hemodynamic variables during expiratory ventilation assistance in a hypovolemic subject. The end-expiratory pressure (EEP) progressively decreases over time. The cardiac output (CO) increases as end-expiratory pressure decreases

Figure 3 shows the hemodynamic response to EVA after hemorrhage. Compared with conventional ventilation with PEEP 0cmH_2_0, subjects treated with EVA of − 6 and − 8 had higher median cardiac output, higher mean systemic arterial pressure, and lower central venous pressure at 40 and 60 min. The median cardiac output was 41% higher in the EVA group than the control group at 60 min [2.70 vs. 1.59 L/min, *p =* 0.029]. During resuscitation, there were no significant hemodynamic differences between the two groups. Figure 4 shows the effect of EVA during hypovolemia in a representative subject. With a stepwise reduction in expiratory pressure, there was a progressive fall in airway pressure, central venous pressure, and pulmonary artery pressure. However, there was a simultaneous progressive increase in left ventricular and mean systemic pressure.

**Fig. 5:**
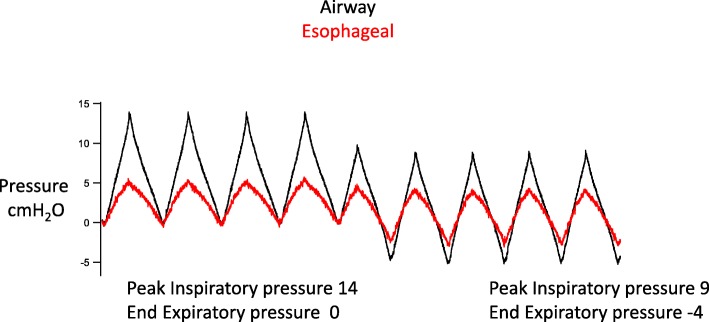
Example of the effect of expiratory ventilation assistance on esophageal pressure

**Fig. 6:**
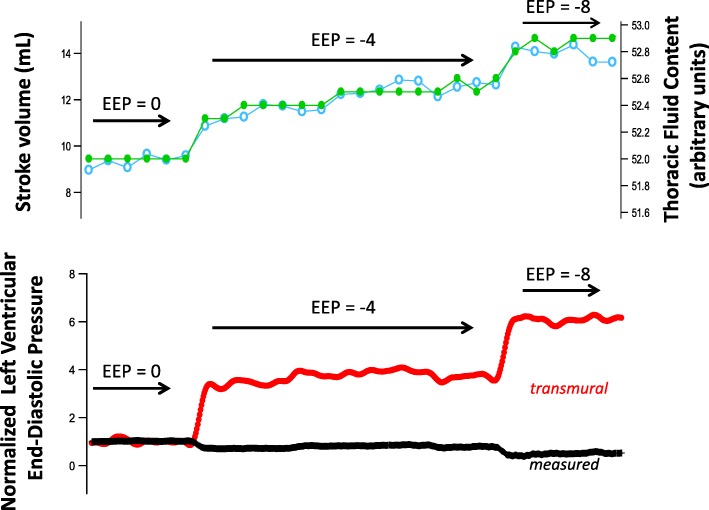
Example of the dose-response of negative end-expiratory pressure on indices of left ventricular filling and ejection. The top panel displays changes in thoracic fluid content and stroke volume measured by bioreactance. The bottom panel shows the change in measured and transmural left-ventricular end diastolic pressure (EDP) as the expiratory pressure decreases. Ventricular pressures were normalized to their baseline values. This demonstrates that while measured EDP declined slightly, transmural EDP increased nearly sixfold

Figure 5 demonstrates in a single subject the effect of EVA on esophageal pressure, which is a surrogate for intrathoracic pressure. The figure suggests that reducing the end-expiratory tracheal pressure with EVA reduces intrathoracic pressure. Figure 6 shows an approximation of the transmural pressure of the left ventricle obtained during progressively lower end-expiratory pressures. The transmural pressure was calculated by subtracting the esophageal pressure from the end-diastolic pressure. The increased end-diastolic transmural pressure of the left ventricle was associated with an increase in thoracic fluid content and stroke volume measured by bioreactance. Thoracic fluid content is a surrogate for cardiac preload.

**Fig. 7:**
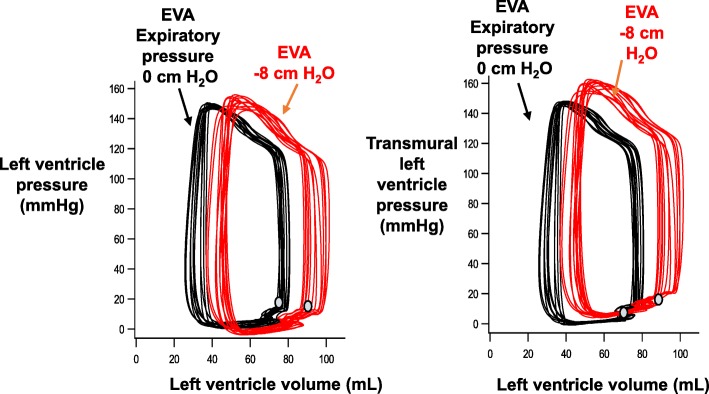
Pressure-volume loops from one subject during expiratory ventilation assistance (EVA). The left panel shows a decrease in measured left ventricular end diastolic pressure (gray circles) as expiratory pressure decreases. The right panel shows the same data, but with an estimate of transmural left ventricle pressure on the *Y*-axis. This demonstrates that negative expiratory pressure with EVA increases left ventricular wall tension

Figure 7 displays the effect of EVA on the left ventricular pressure-volume relationship in a representative subject. The left side of the panel shows that a lower expiratory pressure was associated with an increased end-diastolic volume of the left ventricle and an increase in stroke volume and myocardial work. The right side of the panel uses the estimated transmural pressure of the left ventricle for the *y*-axis of the pressure-volume relationship. The figure suggests that the wall tension of the left ventricle increased with negative expiratory pressure.

## Discussion

This study showed that EVA through a small endotracheal tube increased stroke volume, cardiac output, and mean arterial pressure compared with conventional ventilation. The hemodynamic effects of EVA were most significant in the hypovolemic state after hemorrhage. This study provides additional support for Guyton’s venous return model of the circulation, which asserts that the gradient between the systemic veins and the right atrium powers the return of blood to the heart. The model predicts that negative intrathoracic pressure reduces the absolute right atrial pressure and increases the filling of the right heart. In this scenario, the lower right atrial pressure is matched by an increase in its transmural pressure. This increase in the venous return can increase the left ventricular end-diastolic volume, which can increase stroke volume [5–7, 19]. Several lines of evidence from this report suggest that EVA increased stroke volume by increasing the pressure gradient for venous return. First, the esophageal probe waveforms demonstrate that EVA was able to reduce intrathoracic pressure. Second, EVA reduced absolute right atrial pressure. Third, left ventricular end-diastolic volume and thoracic fluid content increased as the intrathoracic and intra-cardiac pressures decreased. Fourth, the increase in stroke volume and left ventricular end-diastolic volume were proportional to the decrease in expiratory pressure. Finally, it is unlikely that any factor other than an increase in preload improved the stroke volume. EVA did not improve either contractility or decrease left ventricular afterload. While we were not able to measure end-systolic elastance, a surrogate marker of contractility (dP/dt) did not increase. Moreover, the transmural wall tension (afterload) increased with negative pressure exhalation.

This study may have underestimated the potential ability of EVA to improve venous return compared with conventional ventilation during hemorrhagic shock. In clinical practice, conventional ventilation typically uses PEEP, which can diminish the gradient for venous return. In fact, PEEP is fatal in porcine hemorrhagic shock [20]. Therefore, the control arm in this study used an expiratory pressure of 0 cmH_2_0 rather than PEEP.

This study is a preliminary evaluation of a newly automated ventilation device. Theoretically, a clinical application for EVA might be for temporary use during hemorrhagic shock before resuscitation. If technical improvements allow, a clinician could quickly place the Tritube catheter into an endotracheal tube and temporarily use EVA. As soon as hemodynamic crisis resolves or the patient begins to make spontaneous breathing efforts, the catheter could be removed, and conventional ventilation resumed. However, it is essential to consider that despite the compelling physiologic rationale, other negative pressure ventilation strategies were not efficacious in clinical trials [21].

While this study focused on hemorrhage, there is a theoretical benefit of EVA in other hemodynamic states that would benefit from a temporary increase in venous return. Other negative pressure devices have increased cardiac output in patients with Fontan physiology, cardiac tamponade, and cardiopulmonary resuscitation [22–24]. Moreover, by decreasing intrathoracic pressure, other negative pressure devices have increased cerebral drainage and increased cerebral perfusion [24, 25]. The increase in venous return and cerebral perfusion pressure are theoretical advantages for using EVA as a transtracheal rescue ventilation system during cardiopulmonary resuscitation. Conversely, it may be problematic to use EVA as a ventilator strategy during airway surgery in patients with congestive heart failure. These patients are less likely to tolerate increases in preload and left ventricular afterload from EVA.

A significant limitation of the automated EVA system is the potential for obstruction of the thin catheter, which occurred twice during our experiments. A system for flushing the catheter would be helpful. Moreover, a larger diameter endotracheal tube could prevent obstruction. A larger tube might also improve CO_2_ clearance, which is significant because EVA is less effective at ventilation than the conventional strategies. Dead space, as estimated by minute ventilation and PCO_2_, rose slightly upon initiation of EVA. Previous investigations have also shown that EVA is more efficient at oxygenation than carbon dioxide clearance [15].

Aside from the specific limitations of EVA, there are two crucial problems of negative pressure exhalation in general. First, negative pressure exhalation may increase the risk of pulmonary edema in susceptible hosts. This study shows that EVA increases left ventricular preload and may increase left ventricular wall tension. Second, EVA may lead to atelectasis and ventilator-induced lung injury. Negative intrathoracic pressure could lead to atelectrauma and cyclic shear. While short-term extrathoracic negative pressure may be safe, the effects of EVA on the lung are unknown [26, 27]. Moreover, it is unclear how low the expiratory pressure should be set with EVA. In prior studies of negative pressure ventilation during hypovolemia, venous return increased with a decrease in intrathoracic pressure as great as − 10 cmH_2_0. [28]

EVA differs from other negative pressure strategies such as the extrathoracic cuirass ventilator. Unlike EVA, the cuirass expands the thorax, which creates negative intrathoracic pressure to assist inspiration. EVA and other Venturi-assisted ventilators apply negative pressure to the airway to assist exhalation [9, 11]. It is likely that the different types of negative pressure ventilators have different clinical and physiologic effects.

There are some technical limitations of this study. First, this pilot study used a small sample size. Additionally, the esophageal pressures are only surrogates for pleural and intrathoracic pressure. The esophageal pressure is useful for trending, but absolute values are unreliable [29]. Therefore, our calculations of transmural pressure are just estimates. Another limitation is the unreliability of the left ventricular conductance catheter during severe hypovolemia. We were not able to obtain reliable left ventricular volume measurements during hemorrhage for all subjects due to catheter movement and signal artifact produced by turbulent flow.

## Conclusion

EVA through a small endotracheal tube increased venous return, cardiac output, and mean arterial pressure compared with conventional positive pressure ventilation. The effects were most significant during hypovolemia from hemorrhage. EVA provided less effective ventilation than conventional positive pressure ventilation.

## Abbreviations

CVP: Central venous pressure
EEP: End-expiratory pressure
EVA: Expiratory ventilation assistance
FiO_2_: Fraction of inspired oxygen
NICOM: Non-invasive cardiac monitor
PEEP: Positive end-expiratory pressure
